# Effects of Geometry on the Electronic Properties of Semiconductor Elliptical Quantum Rings

**DOI:** 10.1038/s41598-018-31512-4

**Published:** 2018-09-05

**Authors:** J. A. Vinasco, A. Radu, E. Kasapoglu, R. L. Restrepo, A. L. Morales, E. Feddi, M. E. Mora-Ramos, C. A. Duque

**Affiliations:** 1 Grupo de Materia Condensada-UdeA, Instituto de Física, Facultad de Ciencias Exactas y Naturales, Universidad de Antioquia UdeA, Calle 70 No. 52-21, Medellín, Colombia; 2 Department of Physics, “Politehnica” University of Bucharest, 313 Splaiul Independenţei, Bucharest, RO 060042 Romania; 3 Faculty of Science, Department of Physics, Cumhuriyet University, 58140 Sivas, Turkey; 4 Universidad EIA, CP 055428 Envigado, Colombia; 5 Laboratoire de Matière Condensée et Sciences Interdisciplinaires (LaMCScI) Group of Optoelectronic of Semiconductors and Nanomaterials ENSET, Mohammed V University in Rabat, Rabat, Morocco; 6 Centro de Investigación en Ciencias-IICBA, Universidad Autónoma del Estado de Morelos, Av. Universidad 1001, CP 62209 Cuernavaca, Morelos Mexico

## Abstract

The electronic states in GaAs-Al_*x*_Ga_1−*x*_As elliptically-shaped quantum rings are theoretically investigated through the numerical solution of the effective mass band equation via the finite element method. The results are obtained for different sizes and geometries, including the possibility of a number of hill-shaped deformations that play the role of either connected or isolated quantum dots (hills), depending on the configuration chosen. The quantum ring transversal section is assumed to exhibit three different geometrical symmetries - squared, triangular and parabolic. The behavior of the allowed confined states as functions of the cross-section shape, the ring dimensions, and the number of hills-like structures are discussed in detail. The effective energy bandgap (photoluminescence peak with electron-hole correlation) is reported as well, as a function of the Al molar fraction.

## Introduction

During the last decades, the improvement in materials growth techniques has made possible the practical realisation of semiconductor-based nanoscopic structures such as quantum wells, quantum dots and quantum rings (QRs), which have attracted strong technological interest as a result of the possibility of tailoring their electronic and optical properties through the modific and shape^1,2^.

Roughly speaking, the QRs can be considered as quantum dots with a “hole” at the center^3^. The quantum dots have an electronic spectrum similar to that of atoms, hence they are commonly called “artificial atoms”, but they are easily controlled, as opposed to atoms, by changing the geometry and sizes of the artificial structure^4^. The interest towards these particular nanostructures lies in their novel and outstanding electronic, magnetic, and optical properties^5–7^.

Despite the advances achieved in growth techniques such as molecular beam epitaxy and metal chemical vapor deposition, that have allowed to fabricate QRs with geometries close to the circular one -which have been studied by many groups in recent years^8–16^, the actual rings do have a more complex structure due to the presence of impurities and, more importantly, geometrical imperfections^10,17,18^. For instance, the deviations from the ideal toric-shape can be observed from AFM images of QRs obtained via droplet epitaxy^19,20^. Using the same growth technique (that allows varying the ring geometry), Kuroda *et al*. were able to produce concentric double rings^21^. Besides, InGaAs quantum rings can be fabricated by the Stranski-Krastanov growth procedure, where the first step is to get an island and then the ring-shape can be reached^22^.

The effects of intense laser field on the linear and nonlinear electronic related optical properties in quantum rings have been reported. Several geometries such as single and double quantum rings under applied electric and magnetic were studied. The authors report, for instance: (*i*) blue and/or redshift of the optical absorption associated to the incident light polarization^23^, (*ii*) blue and redshift on the optical absorption due to the simultaneous influences of an intense laser field and lateral applied electric fields^24^, and. (*iii*) linear and quadratic quantum confined Stark effects^25^. The adiabatic approach has been intensely used in recent years to describe electronic and impurity states and molecular and excitonic complexes in semiconductor QRs of In_*x*_Ga_1−*x*_As embedded in GaAs medium and GaAs embedded in Al_*x*_Ga_1−*x*_As^26–30^. In the case of self-assembled QRs, the results show that although the real shape differs strongly from an idealized circular-symmetric, the Aharonov-Bohm oscillations of the magnetization survive^26,27^. The study of QRs, with modulated height, shows that: (*i*) the shape has a dominant character with respect to the Coulombic effects on the energy structure, (*ii*) the molecular complex stability is highly sensitive to the number of hills along the axial direction^28^, and (*iii*) the Aharanov Bohm oscillation patterns are very sensitive against changes of the structural parameters and applied electric field, which usually is used to break the axial symmetry of the QR-shaped heterostructures^29,30^.

Thus, it becomes interesting to investigate the elliptically-shaped QRs. Besides, being more realistic, they actually have potential applications, such as gain medium in lasers, medium-infrared and THz range detectors^31–34^. A special use for THz range devices is the detection of explosives at a distance, since the substances that usually compose them have a response at those frequencies^35^. In addition, some studies related to impurities in two dimensional elliptical quantum rings have been carried out focusing on their influence on persistent current features^10,36,37^.

Among many applications of QRs with eccentricity effects it is possible to mention high-density memories and spintronic devices^38^. These elliptically-shaped QRs attract attention because of the features of the Aharonov-Bohm (AB) effect observed and related to the ring topology, being an excellent example of quantum-mechanical phase coherence^39–42^. This quantum mechanical phenomenon has important implications on the denominated persistent electron current^43^. Omidi *et al*. have investigated it with combined effects of magnetic flux and Rashba spin-orbit interaction in elliptical quantum rings^44^. Khordad has studied the Rashba spin-orbit interaction in eccentrical double quantum rings^45^. In fact, those spin-related phenomena are relevant for applications in spintronics^46^. Hence, studies have also included simultaneous effects such as the combined influence of hydrostatic pressure and spin-orbit coupling on linear and nonlinear absorption coefficients in GaAs quantum rings^47^. Additionally, non-equilibrium Keldysh Green’s functions technique have been used in ref.^48^ to investigate transport properties of a system of three quantum dots placed in a ring-like configuration, with prospective applications in quantum switching and efficient spin filtering. Furthermore, a report on the combined effect of temperature and pressure on the electronic and optical properties of excitons in GaAs-based elliptical QRs is currently in press^49^.

Other important studies are related with coupled QRs, in which THz radiation and electric field effects have been analyzed, showing the possibility of tailoring the degeneracy of the discrete energy spectrum as well as the tunneling effect for structures of low dimensionality^50^. In the past, several studies with threading magnetic field effects have concluded that the electron spectrum is sensitive to the competition between curvature and width in QRs, and the reader is referred, for instance, to the work of Bruno-Alfonso and Latgé^51^. A bilayer graphene (BG) eccentrical QR is studied in^52^. The authors claim that such a structure can be applied in the development of electronic devices. This particular QR system has different properties compared with both graphene and graphite since a gapless electronic structure can be tailored by means of created electrostatic potentials. In a very recent report, Shi *et al*. discussed the geometry, electric field, and impurity position effects on the Stark shift and photoionizations cross section for core/shell ellipsoidal quantum dots^53^.

In a very recent report, Bejan *et al*. studied GaAs/AlGaAs elliptic QRs under eccentricity and electric field effects and demonstrated the generation of second and third harmonic optical responses^54^. Besides, a signature of Majorana fermions was found in ref.^55^ for elliptical quantum rings. The energy spectrum in toroidal quantum rings with two different transversal cross section, circular and square, with donor impurity effects was investigated in^56^. A two dimensional Hamiltonian was used to study electron states with magnetic field effects in quantum rings for different eccentricities^57^. Simonin and Barticevic made a comparative study between hill and volcano lateral confining potential in QRs^58^, whilst Mughnetsyan and Kirakosyan used the Green’s function formalism to calculate strain in a InAs/GaAs quantum ring superlattice and to study its effects on the band structure^7^. Also recently, the effects of intense terahertz laser field on electronic and optical properties of QRs were investigated in^59^.

With all this in mind, we have carried out the investigation of the energy states of an electron in elliptical semiconductor QRs. For that purpose we numerically solve the 3D conduction band position-dependent-effective-mass equation in the parabolic approximation via the Finite Element Method (FEM), using COMSOL-Multiphysics comercial software^60^. The aim is to analyse the influence of the size of the structure, the geometry of the ring’s transverse cross section (either squared, parabolic or triangular) as well as to include the possibility of a variable number of hill-shaped deformations along the vertical direction—as appearing in the AFM images mentioned above^19,20^ — that may behave as inserted quantum dots. In accordance, the work is organized as follows: The section II is devoted to the theoretical model. Section III includes the results and the corresponding discussion and, finally, in section IV the conclusions of the work are given.

## Theoretical Model

In this paper we consider a model semiconductor elliptical ring with variable geometry, consisting of a GaAs QR grown on a Al_*x*_Ga_1−*x*_As substrate and embedded in a three-dimensional matrix of the same material. The volume of the QR is delimited by an in-plane elliptical crown (EC) contained in the *xy*-plane and by a differential surface (DS) contained in the *z* > 0 semi-space. The cartesian equation of the EC reads:

1 $$g(x,y,{R}_{x2},{R}_{y2}) < 1 < f(x,y,{R}_{x1},{R}_{y1}),$$

where *f* and *g* describe the inner and the outer borders of the QR which are defined by:

2 $$f(x,y,{R}_{x1},{R}_{y1})={(\frac{x}{{R}_{x1}})}^{2}+{(\frac{y}{{R}_{y1}})}^{2}=1;$$

3 $$g(x,y,{R}_{x2},{R}_{y2})={(\frac{x}{{R}_{x2}})}^{2}+{(\frac{y}{{R}_{y2}})}^{2}=1,$$

where *R*_*x*1_, *R*_*x*2_ are the semi-axes along the *x*-direction of the inner and outer ellipses respectively, and *R*_*y*1_, *R*_*y*2_ are the semi-axes along the *y*-direction.

Considering three points at the same angular position in the plane *z* = 0: (*x*_1_, *y*_1_), (*x*, *y*), and (*x*_2_, *y*_2_), with the first (third) one located in the inner (outer) border of the ring and the second one in the region delimited by the borders, we can define three distances ${\rho }_{1}={({x}_{1}^{2}+{y}_{1}^{2})}^{\mathrm{1/2}}$, $\rho ={({x}^{2}+{y}^{2})}^{\mathrm{1/2}}$, and ${\rho }_{2}={({x}_{2}^{2}+{y}_{2}^{2})}^{\mathrm{1/2}}$, with ${\rho }_{1}^{2}\le {\rho }^{2}\le {\rho }_{2}^{2}$. This relationship between the three radii, combined with Eq. (1), gives ${f}^{-\mathrm{1/2}}={\rho }_{1}/\rho \le 1\le {\rho }_{2}/\rho ={g}^{-\mathrm{1/2}}$. In the case of triangular transversal section of the ring, with sizes *ρ*_2_ − *ρ*_1_ and *h* of the base and height, respectively, is direct to demonstrate that any point located on the ring-boundary, with *z* ≠ 0, is given by $z(\rho )=h[1-|2\rho -{\rho }_{2}-{\rho }_{1}|/({\rho }_{2}-{\rho }_{1})]$. This expression, combined with the relationships between radii, finally gives $z(\rho )=h[1-|2-{g}^{-\mathrm{1/2}}-{f}^{-\mathrm{1/2}}|/({g}^{-\mathrm{1/2}}-{f}^{-\mathrm{1/2}})]$. Following the same arguments, a very similar expression can be obtained by considering parabolic or squared transversal sections of the rings. Summarizing, in order to define the shape of the DS we write the following mathematical expression representing a function of any point (*x*, *y*) ∈ *EC*:

4 $$z(x,y)=h(x,y)[1-{(\frac{|2-{g}^{-\mathrm{1/2}}-{f}^{-\mathrm{1/2}}|}{{g}^{-\mathrm{1/2}}-{f}^{-\mathrm{1/2}}})}^{p}].$$

In this equation *p* = 1, 2, +∞ stand for triangular, parabolic or square radial profiles respectively. *h* represents the angularly modulated height of the quantum ring in *z*-direction which is given by

5 $$h(x,y)=H+A\,\cos \,[n\,\,\arccos \,(\frac{x}{\sqrt{{x}^{2}+{y}^{2}}})]$$

where *H* denotes the reference height of the quantum structure, *A* represents the amplitude of the harmonic modulation, and *n* = 0, 1, 2, 3, 4, … is the number of hill-shaped height maxima. Following Kuroda *et al*., unless explicit reference to the *H* and *A* values, we will be restricting ourselves to *H* = 5 nm and *A* = 2.5 nm^21^.

**Figure 1:**
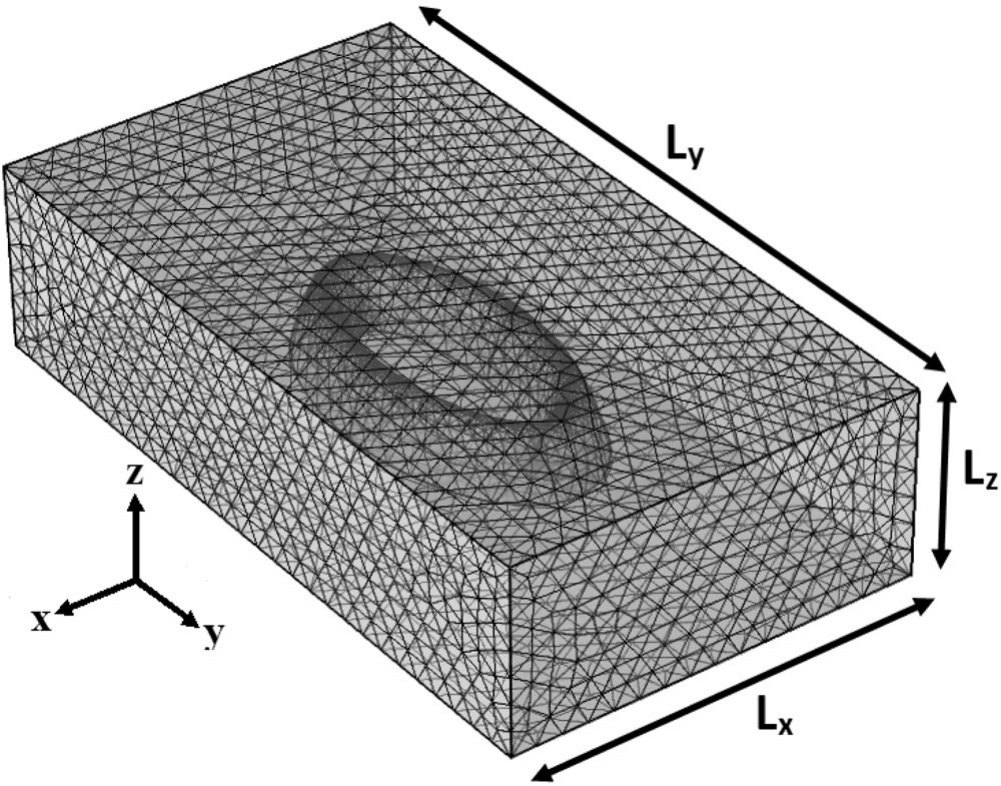
An example of the mesh for the system. *L*_*x*_ = 4*R*_*x*2_, *L*_*y*_ = 4*R*_*y*2_, and *L*_*z*_ = 3(*H* + *A*) represent the parallelepiped dimensions.

From Fig. 1, it is possible to observe an example of the mesh used to solve the Schrödinger equation by using FEM. The building elements are of tetrahedral type, with extra fine and self-adjusted element size for changing ring dimensions. The embedding matrix is represented by a parallelepiped whose edges were used to impose Dirichlet boundary conditions, i.e., the wavefunctions vanish at the edges. An example of used mesh parameters is the following: For QR dimensions *R*_*x*1_ = 25 nm, *R*_*y*1_ = 15 nm, and four hill-shaped deformatios (n = 4), the mesh is built with 112169 tetrahedral elements, 7608 triangular elements, whilst the parallelepiped dimensions are *L*_*x*_ = 130 nm, *L*_*y*_ = 90 nm, and *L*_*z*_ = 22.5 nm.

**Figure 2:**
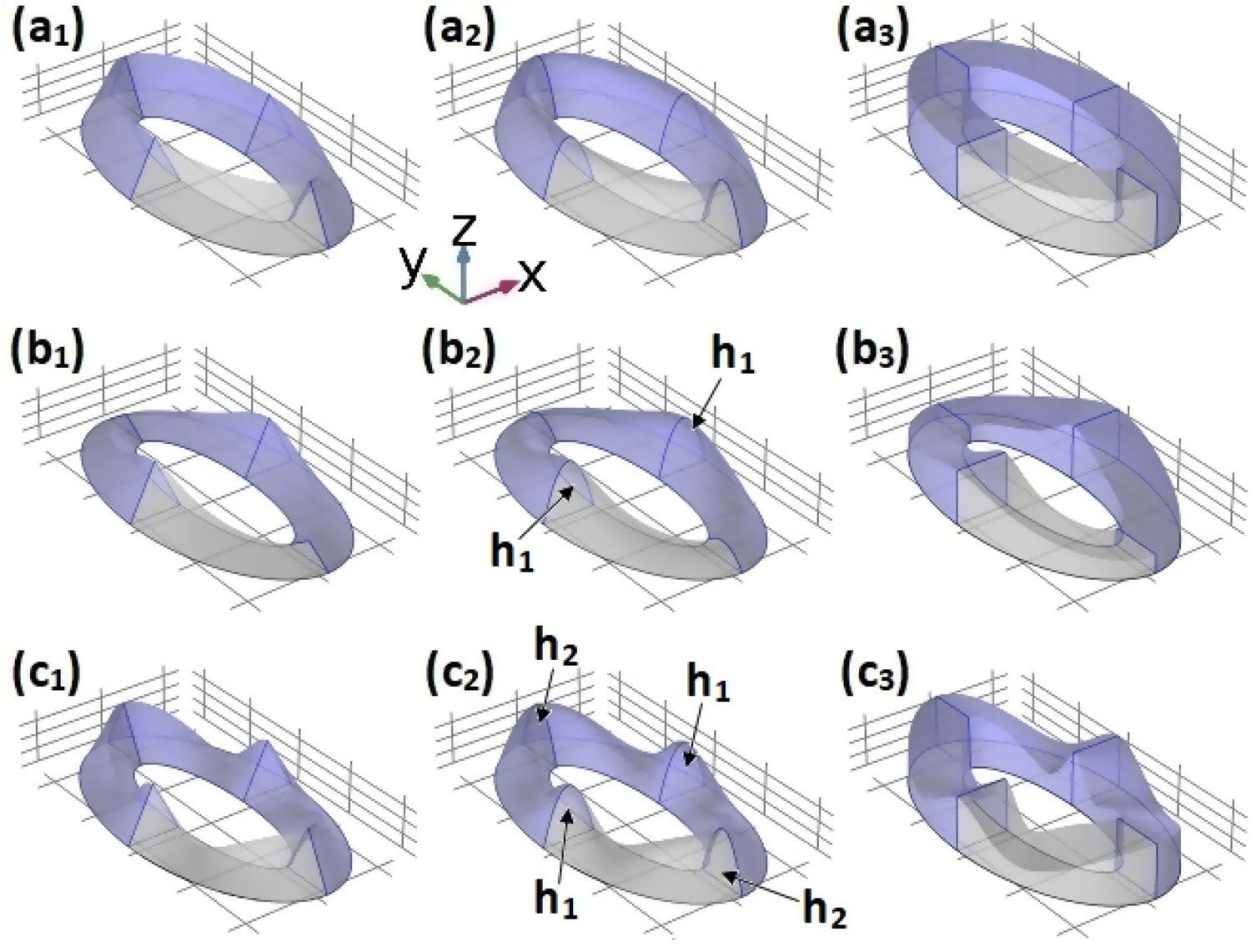
Schematic representation of the elliptical quantum ring structure considered in this work. Three different transversal profiles have been considered: triangular (*p* = 1), parabolic (*p* = 2), and square (*p* → ∞) [see Eq. (4)], corresponding to the first, second, and third columns, respectively. In each row, the number of hills is set as (*n* = 0), two (*n* = 2), and four (*n* = 4), corresponding to the first, second, and third rows, respectively. The *h*_1_ labels in panel (*b*_2_) are used to mark two kinds of identical opposite hills and the *h*_1_ and *h*_2_ labels in panel (*c*_2_) are to mark the two pairs of hills.

In Fig. 2, a schematic representation of the system under study is shown. Two conditions can be observed. On the one hand, when moving through the rows from left to right, one notes a change in the profile of the transversal cross section. On the other hand, when moving through the columns from top to bottom, one may observe a different number of hills. In the panels (*b*_2_) and (*c*_2_), some labels have been placed on the hills in such a way that we can refer to them during the discussion. In (*b*_2_) there are two hills along the *x*-axis, labeled as *h*_1_ and in (*c*_2_) four hill-shaped deformations have been built, two of them on the *x* – axis, labeled as *h*_1_, and the other two on the *y* – axis, denoted as *h*_2_. In the first row (*n* = 0) the ring constant height is *h* = *H* + *A*, whereas in the second and third rows the hills height is *h* = *H* + *A* and the inter-hill regions have minimum height of *h* = *H* − *A* [see Eq. (5)].

**Figure 3:**
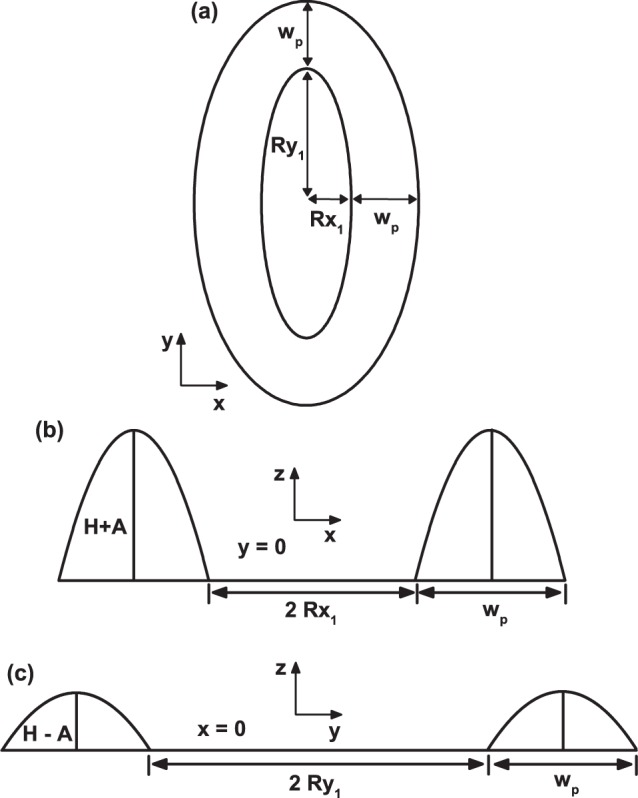
Perspective views of the elliptical quantum ring from Fig. 2(b_2_). In (**a**) a perspective view over the *xy* plane and the length of semi-axes are shown, in (**b** and **c**) the perspective views over the *yz* and *xz* planes are depicted, with their respective maximum (*H* + *A*) and minimum (*H* − *A*) height. The intermediate heights, in the inter-hill regions, are not shown.

In Fig. 3, the graphics corresponding to the panel (*b*_2_) from Fig. 2 has been redrawn to allow noticing the projections of the system on the planes *xy* (a), *xz* (b), and *yz* (c). In Fig. 3(a), *R*_*x*1_ and *R*_*y*1_ correspond to the dimensions of the inner ellipse semi-axes and *w*_*p*_ is the constant separation (with *z* = 0) between the inner and outer ellipse, *i*.*e*., *R*_*x*2_ = *R*_*x*1_ + *w*_*p*_ and *R*_*y*2_ = *R*_*y*1_ + *w*_*p*_. In Fig. 3(b), it is depicted the transversal profile associated to the *h*_1_-region from Fig. 2b_2_ (with base *w*_*p*_ and height (*H* + *A*). Also, the separation between the two hills (2*R*_*x*1_) is shown. In Fig. 3(c), the profile corresponds to the two lowest transversal area (with base *w*_*p*_ and height *H* − *A*) with 2*R*_*y*1_ being the separation between the two inner extremes.

The 3D Hamiltonian in the framework of the effective mass and parabolic band approximation is given by:

6 $$H=-\,\,\frac{{\hslash }^{2}}{2}\nabla \cdot (\frac{1}{{m}^{\ast }}{\nabla }_{x,y,z})+V(x,y,z),$$

where the values of m^*^ and *V*(*x*, *y*, *z*) depend on the region where the Schrödinger equation is solved. The conduction effective mass is taken to be position-independent within each of the composing materials: ${m}^{\ast }={m}_{in}^{\ast }=0.067\,{m}_{0}$ (GaAs), and ${m}^{\ast }={m}_{out}^{\ast }=0.092\,{m}_{0}$ (Al_0.3_Ga_0.7_As), where *m*_0_ is the free electron mass^21^. Besides, the potential energy function, *V*(*x*, *y*, *z*), is defined as zero within the inner region of the QR (GaAs) and value of *V*_0_ elsewhere.

The distance between the two elliptical borders is kept fixed. For it we choose the value *w*_*p*_ = 7.5 nm [see Fig. 3]. As mentioned, the solution of the Schrödinger-like effective mass equation for the Hamiltonian in Eq. (6) was sought using the FEM, where boundary conditions were imposed in the following way: (1) wavefunction continuity on the border of the QR, *ψ*_*in*_ = *ψ*_*out*_ (2) continuity of $\frac{1}{{m}^{\ast }}{\nabla }_{x,y,z}\psi$ at the GaAs-Al_*x*_Ga_1−*x*_As interface ($\frac{1}{{m}_{in}^{\ast }}\frac{\partial {\psi }_{in}}{\partial X}=\frac{1}{{m}_{out}^{\ast }}\frac{\partial {\psi }_{out}}{\partial X}$ with *X* = *x*, *y*, and *z*), and (3) Dirichlet boundary conditions on the matrix (Al_*x*_Ga_1−*x*_As) border, *ψ* = 0. This method was revealed as a powerful tool to solve problems with complicated geometry^61^.

## Results and Discussion

**Figure 4:**
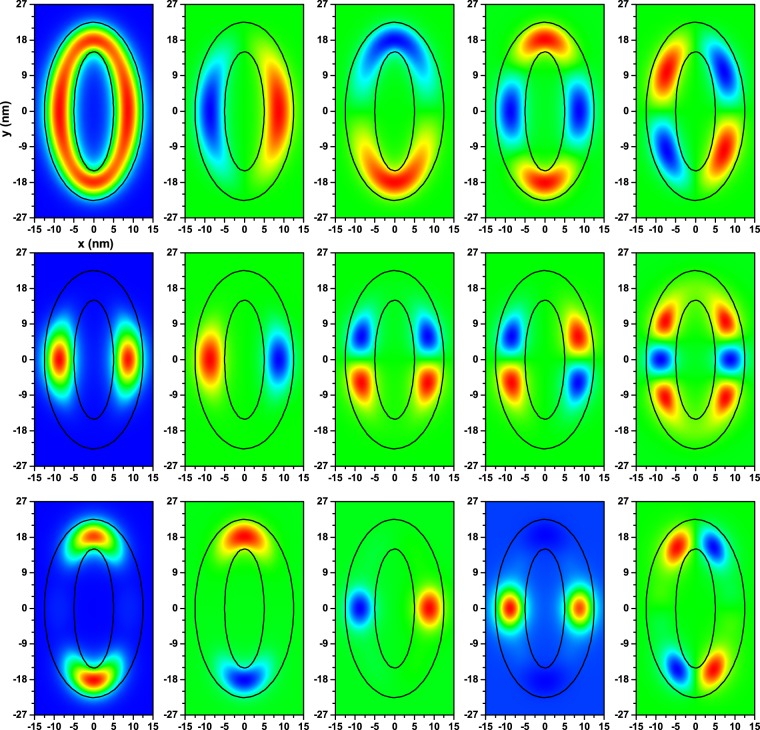
The *z* = 3.75 nm projections, of the electron wavefunctions for the first five confined electron states in an elliptical quantum ring with parabolic transversal section (from left to the right, the columns correspond to the electron wavefunctions *ψ*_*k*_ with *k* = 1, 2, 3, 4, and 5). The first, second, and third rows are for *n* = 0, 2, and 4, respectively (see Eq. (5) and the second column in Fig. 2). Results are obtained for *R*_*x*1_ = 5 nm and *R*_*y*1_ = 15 nm. The ellipses in black color correspond to the quantum ring profile projected onto the plane *xy* (*z* = 0). The colors mean the following: in the first column blue is zero, and red is the maximum positive value (the same is for the panel localized at the third row combined with the forth column). In the rest of the figure blue-green-red represent negative, zero, and positive values, respectively.

The Fig. 4 contains the projections of the first five state wavefunctions onto the *z* = 3.75 nm plane, for an electron confined in an elliptical QR with parabolical transversal section, for several values of the number of hills, and fixed QR dimensions. Note that the *z* = 3.75 nm plane corresponds to the mid-height for the case *n* = 0, where the height of the ring is uniform, and essentially determines the position where the probability density of electrons reaches its maximum value. The results are presented for distinct numbers of hill-like vertical deformations, namely *n* = 0, *n* = 2, and *n* = 4; for the first, second, and third row, respectively. By observing the first row, corresponding to a uniform elliptic-shaped structure, one may note that for the ground state (*ψ*_1_, which has *s*-symmetry) the largest localization of the electron (associated with the maximum of the probability density) occurs for positions close to the vertical and horizontal extremes of the elliptical region, with larger values along the minor axis (*x*-axis in Fig. 4). For levels *ψ*_2_ and *ψ*_3_, also the maximum localization of the electron corresponds to regions on the semi-axes extremes. The states *ψ*_2_ and *ψ*_3_ have *p*_*x*_ and *p*_*y*_ symmetry, respectively, and they are quasi-degenerate. The states *ψ*_4_, *ψ*_5_ are also quasi-degenerate, and display inversion symmetry; that is, *ψ*_*i*_(*x*, *y*) = *ψ*_*i*_(−*x*, −*y*) (*i* = 4, 5). Clearly, if we had a circular QR, the wavefunctions for the states *ψ*_2_ and *ψ*_3_ (*ψ*_4_ and *ψ*_5_) would be degenerate and equivalent through a rotation of 90° (45°).

In the second row of the figure, the ring exhibits two hill-like regions localized at the two extremes of the smaller-ring semi-axis (named as *h*_1_ in Fig. 2(b_2_)). In the third row the structure has two hills, with larger volume, placed at the two extremes of the bigger semi-axis [named *h*_2_ in Fig. 2(c_2_)], and two hills with smaller volume at the two extremes of the smaller semi-axis [named *h*_1_ in Fig. 2(c_2_)]. Consequently, for *n* = 2 the ground state is two-fold degenerate, and since the two hills are almost isolated from each other, also the first excited state is two-fold degenerate. For the *n* = 4 case, due to the low wavefunction penetration into the inter-hill regions, the ground state and the first excited state are also two-fold degenerate, with the former (latter) associated to the probability of finding the electron in the *h*_2_ (*h*_1_) regions of the hill.

**Figure 5:**
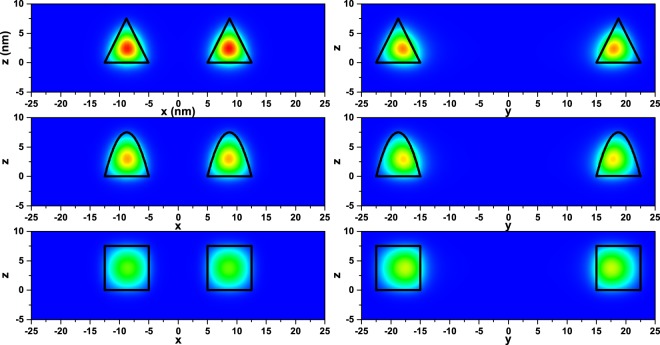
The *y* = 0 projection of the ground state probability density (left column), and the corresponding *x* = 0 projection (right column); for a confined electron in an elliptical QR with *n* = 0, *R*_*x*1_ = 5 nm, and *R*_*y*1_ = 15 nm. The first, second, and third rows correspond to triangular (*p* = 1), parabolic (*p* = 2), and squared (*p* → ∞) transversal sections of the ring, respectively. The blue color means zero and the probability grows positively towards the red color.

The *xz* (*y* = 0) and *yz* (*x* = 0) projections of the ground state probability density (|*ψ*_1_|^2^) of an electron confined in an elliptical QR are shown in Fig. 5 for three distinct ring transversal sections. In the *y* = 0 projection, the maximum of |*ψ*_1_|^2^ lies close to the geometrical center of the profile, showing almost azimuthal symmetry with small deformations associated with the shape of the potential barriers. That is, |*ψ*_1_|^2^ adapts itself to the particular transversal section contour of the ring. Going from the upper to the lower panels and from the left to the right column, one may observe that |*ψ*_1_|^2^ clearly decreases its magnitude. In the first case, the behavior is due to the the increasing transversal section area which causes the wavefunctions to spread over a larger region. In the second case, the observed results are a consequence of the increase in the effective confinement potential. Besides, from the right-hand column it can be observed that the probability densities are shifted towards the inner wall of the transversal section. These are evidences of the *s*-symmetry for the ground state.

**Figure 6:**
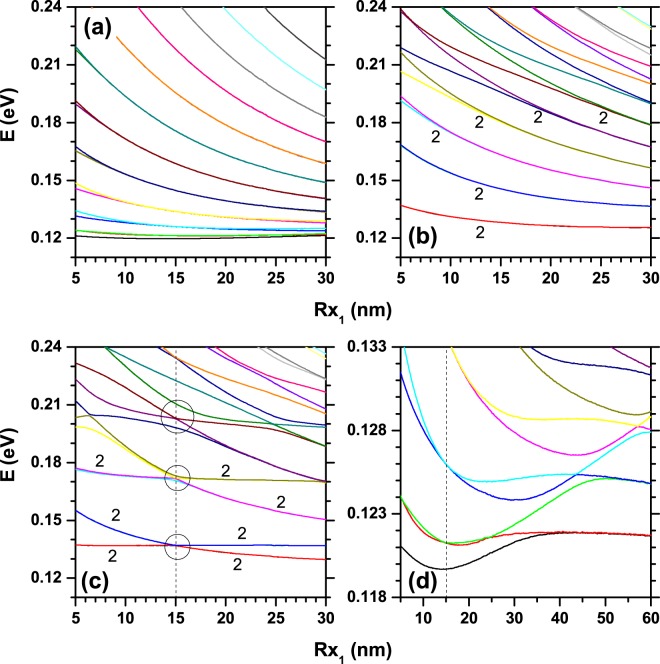
Energy levels of an electron confined in an elliptical QR as functions of *R*_*x*1_, keeping constant *R*_*y*1_ = 15 nm for transversal parabolic profile. Different numbers of hills have been considered: (**a**) *n* = 0 (constant height of the ring such as in Fig. 2(a_2_)); (**b**) *n* = 2 (such as in Fig. 2(b_2_)), corresponding to a pair of equivalent and opposite hills, denoted by symbol “*h*_1_”; and (**c**) *n* = 4 (such as in Fig. 2(c_2_)), corresponding to two pairs of equivalent and opposite hills, denoted by symbols “*h*_1_” and “*h*_2_”. In panel (d) there are shown the first ten lowest energy levels corresponding to the same configuration of panel (a) but with 5 nm ≤ *R*_*x*1_ ≤ 60 nm.

In Fig. 6, the energy levels of an electron confined within an elliptical QR are shown as functions of *R*_*x*1_. The number of hills in the quantum ring have been taken as *n* = 0 (a), *n* = 2 (b), and *n* = 4 (c). Calculations are for *R*_*y*1_ = 15 nm with parabolic transversal cross section. The volume (in nm^3^) of each structure [Fig. 6(a–c)] was adjusted to a quadratic function given by ${\mathscr{V}}=-\,0.01\,{R}_{x1}^{2}+118\,{R}_{x1}+2647$, ${\mathscr{V}}=-\,0.01\,{R}_{x1}^{2}+100\,{R}_{x1}+1446$, and ${\mathscr{V}}=0.1\,{R}_{x1}^{2}+75\,{R}_{x1}+1795$, respectively. Note that the volume of the ring shaped heterostructure was obtained by direct numerical integration using the regular mesh provided by the COMSOL-Multiphysics software^60^. In the particular case of zero hills and squared transversal section, the numerical results of the volume were confirmed with the exact analytical solution.

In general, Fig. 6(a) shows a decreasing behavior of the energies *E*_*i*_ as functions of *R*_*x*1_, due to the growth in volume and, therefore, the reduction in carrier confinement. Note that for *R*_*x*1_ = 5 nm, ${\mathscr{V}}=3236$ nm^3^ while for *R*_*x*1_ = 30 nm, the volume is ${\mathscr{V}}=6174$ nm^3^. The Fig. 6(d) presents a zoom of the 10-lowest energies appearing in Fig. 6(a), with the interval of variation of *R*_*x*1_ extended up to 60 nm. From this panel we can observe a minimum for the ground state of *R*_*x*1_ = 15 nm, whose geometry corresponds to a circular quantum ring. This minimum appears because when *R*_*x*1_ > 15 nm the system actually evolves from a 3D to 2D confinement. This can be verified in the limit of *R*_*x*1_ → ∞, which is observable here for *R*_*x*1_ = 60 nm. In this limit, the ground state is degenerate and corresponds to two parallel uncoupled quantum wires with parabolic transversal section. The verification of this result was achieved with the calculation of the spectra associated with a confined electron into a single quantum wire with the parabolic transversal section, height 7.5 nm and length 60 nm, in which case the value obtained from the ground state is 0.12174 eV. This value is in excellent agreement with the energies of the ground state for the QR studied here [0.12168 eV, see Fig. 6(d)]. Also from Fig. 6(d) one may notice that for *R*_*x*1_ = 15 nm -indicated by a vertical dashed line-, the second and third states are degenerated (*p*_*x*_ and *p*_*y*_ states) due to the circular geometry of the QR. For excited levels, the minimum observed in the ground state -associated to the transition from 3D to 2D confinement- appears as well; but it shifts towards higher values of *R*_*x*1_. This happens due to the 2D confinement regime where the QR wavefunctions are more spatially extended.

In comparison with Fig. 6(a), higher values of the energies are observed in Fig. 6(b). This is related with the lower volumes of these QRs that result in greater electron confinement. For this system with two hills, as shown in Fig. 2(b_2_), a degeneration of the first levels arises, because the two identical hill-like structures behave as isolated regions, i.e., two almost noninteracting hills. In contrast, for higher energies, there is an increasing probability of finding the electron in the lower height parts of the QR (which connect the hill regions). In this case the system behaves like two interacting hills and the degeneracy is lifted for small values of *R*_*x*1_. As *R*_*x*1_ increases, the degeneracy is recovered. These degenerations are indicated by the label “2” in Fig. 6(b).

It is possible to see that the energy spectrum shown in Fig. 6(c) has its lowest levels degenerate, due to the effective division of the ring in four hills (denoted by “*h*_1_” and “*h*_2_” in Fig. 2(c_2_)) that communicate through lower height regions. In these structures, we have two pairs “*h*_1_” and “*h*_2_” of identical hills which correspond to the opposite maximum heights. We can observe a symmetry-exchange between the first and second levels because of -for *R*_*x*1_ < 15 nm- the “*h*_2_”-region has a larger volume as compared to the “*h*_1_”-region (${{\mathscr{V}}}_{a} > {{\mathscr{V}}}_{b}$). That is, the ground state corresponds to an electron localized in “*h*_2_” whereas the first excited states is for electrons in “*h*_1_”. For *R*_*x*1_ > 15 nm, ${{\mathscr{V}}}_{b} > {{\mathscr{V}}}_{a}$ and the ground state corresponds to electrons in “*h*_1_”-region. In the particular case of a circular quantum ring (*R*_*x*1_ = 15 nm), it is observed a four-fold degenerate ground state, since “*h*_1_” and “*h*_2_” regions have the same volume and are practically isolated in the ground state case whilst they are only quasi-isolated for excited states. The circles in Fig. 6(c) show how the degeneracy is lifted as we look up to the higher orders of the excited states. However, it should be noticed that, independently of the order of the levels, there are always two degenerate states, in the indicated circles.

**Figure 7:**
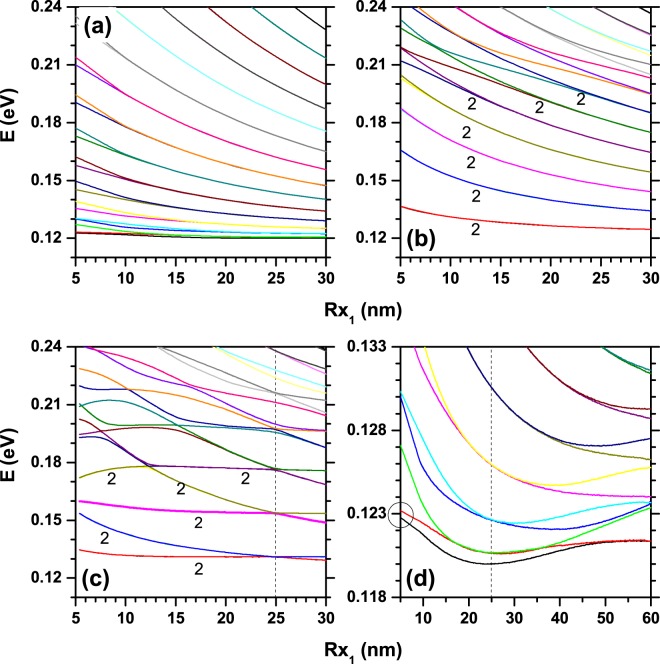
Results are as in Fig. 6, but for *R*_*y*1_ = 25 nm.

In Fig. 7, it is presented the electronic structure for a confined electron in an elliptical QR as a function of *R*_*x*1_, while keeping constant *R*_*y*1_ = 25 nm. The results follow the same geometrical configurations as in Fig. 6. Clearly, the volumes of the QRs in Fig. 7 are larger than the corresponding values in Fig. 6 (note that in Fig. 7 *R*_*y*1_ = 25 nm, whereas in Fig. 6 *R*_*y*1_ = 15 nm). This manifests itself in a lower spatial carrier confinement. Bearing in mind that the vertical scales for energies in Figs 6 and 7 are the same, one may notice that the number of depicted states in panels 7(a–d) is larger than the corresponding one in panels 6(a–d). This means that the increase of *R*_*y*1_ induces a general red-shift of the energy levels and also a smaller inter-level spacing. In Fig. 7(a), as in Fig. 6(a), it is observed a weak dependence of the lowest levels with the QR volume, owing to the concentration of these states in the extreme regions. For small values of *R*_*x*1_, the ground state has a similar behavior to that of two isolated hills localized on the *x*-extremes of the ring, which is a consequence of the large eccentricity. This is evidenced by the tendency towards quasi-degeneration between the first two levels (in panel (d), highlighted by the circular region on the left side; note how the ground state and the first excited one approach the same energy value as *R*_*x*1_ decreases). Extending the comparison between the results in Figs 6 and 7; in the latter the lifting of degeneracies is observed for larger values of *R*_*x*1_ due to the higher structural volume. In addition, some peculiarities are exhibited by the ground state. In Fig. 6, for *R*_*x*1_ = 5 nm and *R*_*y*1_ = 15 nm the ground state energy is *E*_1_ = 121 meV, whereas in Fig. 7, *R*_*x*1_ = 5 nm and *R*_*y*1_ = 25 nm the ground state energy is *E*_1_ = 122.8 meV. In spite of the larger value of the QR volume, for the system considered in Fig. 7 the ground state is shifted to higher energies because the pair of hills localized along the *y*-axis are almost isolated from each other, with a strong spatial localization of the electron wavefunction.

**Figure 8:**
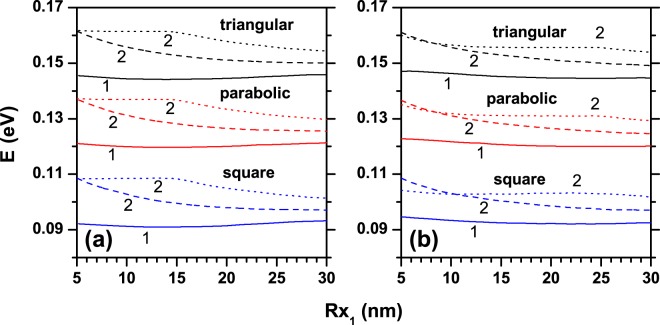
The ground state energy level of a confined electron in an elliptical quantum ring as a function of *R*_*x*1_, for different transversal section profiles (triangular, parabolic, and square). The solid, dashed, and dotted lines correspond to *n* = 0, *n* = 2, and *n* = 4, respectively (see Eq. (5)). *R*_*y*1_ is fixed to: 15 nm (**a**) and 25 nm (**b**). Labels 1 and 2 below the curves correspond to the degree of degeneracy of the level.

The Fig. 8 contains the numerical findings for the *R*_*x*1_ dependence of the ground state energy for an electron confined in an elliptical QR, considering three kinds of transversal section profiles. Two values of the *R*_*y*1_-axis have been taking into account: (a) 15 nm, and (b) 25 nm. The number of hills is represented by solid, dashed, and dotted lines, that correspond to *n* = 0, *n* = 2, and *n* = 4, respectively. The results in these graphics show that as the area of the transversal section increases the curves exhibit almost a rigid shifting to lower energy values (except for the solid line and positive ellipticity, since it evolves from 3D to 2D confinement, *i*.*e*., there is an increase in the allowed energy), which is in accordance with the volume increase. Note that for a specific angular position of the transversal section the area of the squared section is 1.5 times the area of the parabolic section and 2.0 times of the triangular one. Clearly, when the transversal section of the ring evolves from triangular to squared section, passing through the parabolic one, the volume increases and the confinement of the carriers weakens. Note that the considered transversal section shapes do not modify the degree of degeneracy (single or two-fold) for the ground state. Actually, for the ground state, and in the cases of *n* = 2 and *n* = 4, when the transversal section shape is modified, the hills remain isolated and there is a very poor penetration of the electronic probability density into de inter-hill regions. By comparing Fig. 8(a,b) it is possible to observe that in Fig. 8(b) each set of curves (for example the three curves corresponding to the triangular transversal section) occupies a smaller energy interval. This effect is observed mainly in the regime of small radii (*R*_*x*1_ close to 10 nm), and is consistent with the increase in volume associated with higher values of *R*_*y*1_. Furthermore, the Fig. 8(b) shows an interesting effect that becomes apparent when comparing the cases having two and four hill-like regions: In the regime *R*_*x*1_ < 10 nm, a crossing appears between the curves for *n* = 2 and *n* = 4, i.e., when *R*_*x*1_ < 10 nm the energy for *n* = 4 is lower than the energy for *n* = 2, whereas when *R*_*x*1_ > 10 nm the opposite situation occurs.

**Figure 9:**
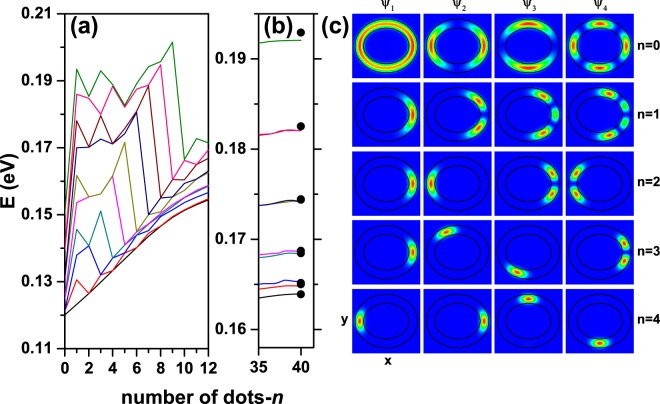
The lowest ten energy levels of a confined electron in an elliptical quantum ring as functions of the number of hill-like regions [*n*, see Eq. (5)] in the case of parabolic transversal section. The number of hills has been taken to vary as 0 ≤ *n* ≤ 12 (**a**) and from 35 ≤ *n* ≤ 40. (**b**) Results are computed for a fixed geometry with *R*_*x*1_ = 20 nm and *R*_*y*1_ = 15 nm. In (**b**) the full dots symbols correspond to the theoretical findings for the ten lowest states in an elliptical quantum ring with parabolic transversal profile and constant height of 4.3 nm. In panel (c) the *z* = 0 projection of the probability density for the first four confined states (the columns are for *ψ*_*i*_, *i* = 1, 2, 3, and 4) considering different numbers of hills (rows from top to bottom correspond to *n* = 0, 1, 2, 3, and 4).

To further analyse the effect of the varying number of hills-like regions, in Fig. 9, the lowest ten energy levels of the confined electron in a parabolic transversal section elliptical QR, appear plotted as functions of the number *n* [Fig. 9(a)]. The largest energy increase occurs for *n* changing from zero to one, since in this case the ring volume decreases from 5000 nm^3^ to 3333 nm^3^. For *n* = 2, the ring volume increases to 3434 nm^3^ and after that the volume remains almost constant and equal to 3334 nm^3^. The first row in Fig. 9(c) shows that the probability density of the first four states distributed over the whole ring-heterostructure. For *n* > 0 the ground state energy is an increasing function of the hill-number due to the reduction of the local volume where the electron is mainly localized. For *n* = 2*k*(*k* = 1, 2, 3, …) the ground state is two-fold degenerate and the maximum of the probability density corresponds to positions close to *x* = ±(*R*_*x*1_ + *R*_*x*2_)/2 (see the first and second columns in the corresponding third and fifth rows). For *n* = 1 there is no degeneracy of states because *x*-symmetry of the potential is broken. In this case, note that the probability density of the depicted states and the profile of the quantum ring have the same reflection symmetry with respect to the *y* = 0 plane. For *n* = 3 the lowest three energy levels correspond to the ground state associated to the electron localized near to *θ* = 0 (*θ* = arctan(*y*/*x*)) [see first column in fourth row of Fig. 9(c)], and two degenerate excited states for the electron in the region *θ* = 2*π*/3, and 4*π*/3 (see second and third columns in fourth row of Fig. 9(c), respectively). The analysis of the other states and number of hills follows the same arguments. In addition, in Fig. 9(b) we present the limit of the energy levels for large *n* values. The full dot-like symbols correspond to the calculation for a QR with parabolic cross section, constant height (*h* = 4.3 nm), and *n* = 0. They represent the energies and the degree of degeneracy for the *n*-asymptotic limit of the curves. In the limit of a high number of hills — Fig. 9(b) —, the energy varies very slowly, because the structure behaves as if there are no hills and the volume reaches its saturation value. Likewise, for higher levels (excited states), and in the range of large *n*, it is readily apparent that the energy has a weak dependence as a function of *n*, because the electron localization is extended over several hills, generating similar probability density to the case of zero hills. In this case the main difference comes from the fact that there is a smaller effective volume, so higher energies are observed than in the zero hill case.

**Figure 10:**
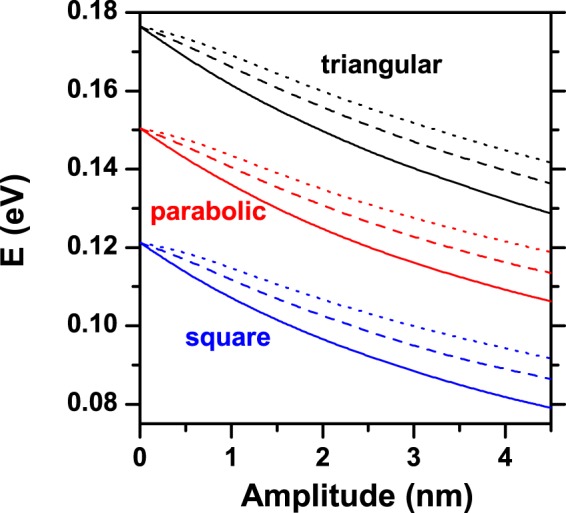
The ground state energy level of a confined electron in an elliptical QR as a function of the height amplitude [see the definition of *A* in Eq. (5)]. Results shown correspond to *R*_*x*1_ = 20 nm and *R*_*y*1_ = 25 nm. Calculations include QRs with triangular, parabolic, and squared transversal sections; several values of the number of hills have been taken into account: *n* = 0 (solid lines), *n* = 2 (dashed lines), and *n* = 4 (dotted lines).

In Fig. 10 we compare the calculated ground state energy of a confined electron in an elliptical QR as a function of the ring’s height amplitude, for the three different cross sections considered in this work. Three values of the *n*-parameter have been considered. By comparing the different transversal geometries, it is clear that the energy diminishes when the area increases by going from the squared profile to the triangular and passing through the parabolic one. This is consistent with the decreasing of energy associated to the growth of the volume in the structure. When *A* → 0, we actually have an angularly constant height because *h* → *H* [see Eq. (5)] Therefore, the energies for zero, two, and four hills converge to the same value. According to Eq. (5), for *n* = 0, the QR height is *h* = *H* + *A*, so the ring’s volume is an increasing function of *A* and, consequently, the energies are decreasing functions of this quantity. For *n* = 2 and 4, the volume of the QR is almost independent of *A*. However, the volume of the regions associated to each isolated hill grows with *A*. This behavior explains the particular decreasing of the energy in these two cases although the whole volume of the ring remains constant.

**Figure 11:**
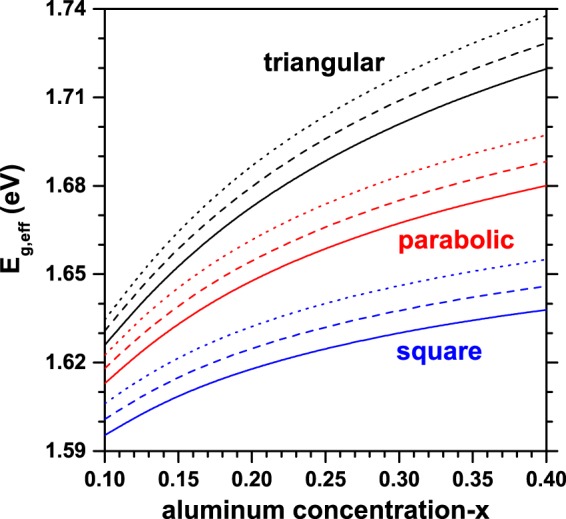
Effective energy gap (photoluminescence peak energy, considering the electron-hole Coulomb correlation) for an electron-hole pair confined in an elliptical QR as a function of the aluminum concentration (*x*) in the barrier material, for the three different transverse profiles (triangular, parabolic and square). Results were computed for *R*_*x*1_ = 20 nm and *R*_*y*1_ = 15 nm. Calculations include QRs with triangular, parabolic, and squared transversal section; several values of the number of hills have been taken into account: *n* = 0 (solid lines), *n* = 2 (dashed lines), and *n* = 4 (dotted lines).

Finally, in Fig. 11, we present the results for the effective energy bandgap–*E*_*g*,*eff*_ (photoluminescence peak energy, with electron-hole correlation)^62–64^ for an electron-hole pair confined in an elliptical GaAs-Al_*x*_Ga_1−*x*_As QR as a function of the aluminum concentration in the barrier material. The results are for different transversal sections of the ring and three values of the *n*-parameter, keeping constant the size of the structure. The effective energy bandgap is defined by *E*_*g*,*eff*_ = *E*_*g*_ (GaAs) + ${E}_{1}^{e}+{E}_{1}^{hh}+{E}_{C}$, where *E*_*g*_ (1.519 eV) is the GaAs bulk bandgap energy at *T* = 4 K and *E*_*C*_ is the Coulomb correlation between the carriers, which has been calculated by using a first order perturbation method. Also, ${E}_{1}^{e}$ and ${E}_{1}^{hh}$ are the electron and heavy-hole ground state energy levels, respectively^65^. In this work, the relation 60/40 for the band offset was used. The *x*-dependence of the electron confinement potential in eV (with *x* < 0.4) is given by ${V}_{0}^{e}=0.873\,x$^66^. The corresponding value for heavy-holes is ${V}_{0}^{hh}=0.582\,x$. The electron and heavy-hole effective masses are, respectively, ${m}_{e}^{\ast }=(0.067+0.084x){m}_{0}$ and ${m}_{hh}^{\ast }=(0.51+0.20x){m}_{0}$^67^, where *m*_0_ is the free electron mass. Note that a spherical heavy-hole effective mass has been assumed. For fixed values of *x*, as it was previously discussed, the electron ground state energy increases (decreases) when the transversal area of the hill (*n*-parameter) decreases (increases). In the case of the heavy-hole, due to its large effective mass value, the effective Bohr radius is of the order of 2–3 nm. Consequently, the heavy-hole ground state is almost independent of the size of the transversal area and of the number of hills. For this reason, the behavior presented by the effective gap, follows the same one associated to the electron ground state. The electron and hole confinement potentials are increasing functions of *x*. It means that as *x*-grows, the electron and hole are more bounded to the ring region. Such aspect manifests itself in a growing character of the electron and hole ground state energies. This variation is more noticeable in the case of the electron given its lower effective mass value. Again, the increasing character of the effective gap as the aluminum concentration increases can be associated to the variation exhibited by the electron ground state.

The perturbative calculations show that the electron-hole Coulomb correlation is in the range of (5, 9) meV for the all reported heterostructures, with the maximum value (9 meV) for the triangular one, with four hills and *x* = 0.4, whereas the minimum value (5 meV) corresponds to the squared quantum ring with zero hills and *x* = 0.1. Note that these calculations confirm the range of validity of the approach considered by Kuroda *et al*. in their work on optical transitions in quantum ring complexes where, due to the dimensions of the structures, they have neglected the Coulomb corrections^21^. Certainly, Coulombic interaction can be introduced analytically in those cases where it is dominant over the effects of confinement^68^. The analytical solutions for a system of two particles subject to Coulomb interaction in 2D and 3D space are well-known. Similarly, the electrostatic interaction between carriers can be introduced with self-consistent techniques combined with the adiabatic approach^69^. Variational techniques with trial functions of one, two, and more variational parameters have proven to be useful in problems of less complexity, for example, in the case of impurities confined in low dimensionality systems^70,71^. In any case, the degree of complexity of the problems can be reduced with an appropriate choice of the coordinate system that takes into account the symmetries of the heterostructures. Summarizing, the effective gap in the elliptical quantum ring follows the behavior of the electron ground state in aspects such as: (i) the shape of the transversal cross section, (ii) the number of quantum hills, and (iii) the aluminum concentration in the barrier material.

Fomin *et al*.^26^ studied the electron-hole energy levels of self-assembled QR under applied magnetic field with and without strain effects. Their study was made in the effective mass approximation and the adiabatic approach. At zero magnetic field, their results follow the same trends of the findings we report here, mainly in the case where we consider two hills along the angular direction. Actually, due to the structural differences between GaAs-InGaAs and GaAs-GaAlAs QR, it is not possible to make numerical comparisons beyond the trends shown by the results. However, it is worth commenting that the working approach presented here, based on numerical calculations through the FEM, provides an excellent alternative to describe the electronic structure -with a high degree of detail- in quantum dots and rings with three-dimensional confinement. A 3D study of the results presented by Fomin *et al*.^26^ in GaAs-InGaAs is currently in process and will be published elsewhere.

## Conclusions

The present work has dealt with the theoretical analysis of the features that characterise the conduction band states of an electron in elliptically shaped quantum rings. The study takes into account the effect of the size and geometry -including the presence of non-uniformities in the form of hill-like structures that behave as quantum dot regions. The theoretical investigation is supported by the numerical solution of the effective mass 3D Schrödinger equation with the use of the finite element method.

The outcome for the electron spectrum confirms the general decreasing behavior - related with the spatial confinement of the charge carriers- of the allowed state energies as functions of the sizes, which is typical of the low-dimensional structures. It is also shown that the change in the geometrical shape of the transversal cross section of the elliptical quantum ring quantitatively affects the spectrum of allowed electron energies. For instance, the results show that higher energy values are associated to the triangle-like quantum ring transversal cross section whilst the smallest correspond to the squared geometry.

On the other hand, we have shown that the presence—or absence—of hill-like regions affects the values of the energy as well but, most of all, it brings along a significant qualitative modification of the set of allowed quantum states and their corresponding energies, which is confirmed by the calculation of the effective energy gaps in the quantum ring. So, the possibility of choosing a particular number of these built-in quantum dot (hill) regions may have an effect on the prospective applications of this kind of system in electronics and optoelectronics.

## Data Availability

All the files with tables, figures, and codes are available. The corresponding author will provide all the files in case they are requested.
